# Antipsychotics-induced hyperprolactinemia and screening for macroprolactin

**DOI:** 10.11613/BM.2021.010707

**Published:** 2020-12-15

**Authors:** Nedjeljka Ruljancic, Ana Bakliza, Sandra Vuk Pisk, Natko Geres, Katarina Matic, Ena Ivezic, Vladimir Grosic, Igor Filipcic

**Affiliations:** 1 Department of Laboratory Diagnostics, Psychiatric Hospital “Sveti Ivan“ Zagreb, Croatia; 2 Faculty of Dental Medicine and Health, “Josip Juraj Strossmayer” University of Osijek, Osijek, Croatia; 3 Department of Integrative Psychiatry, Psychiatric Hospital ‘Sveti Ivan’ Zagreb, Croatia; 4 School of Medicine, University of Zagreb, Zagreb, Croatia

**Keywords:** biochemistry, hyperprolactinemia, macroprolactin, prolactin

## Abstract

**Introduction:**

High prolactin (PRL) concentrations are found in laboratory test results of patients on majority of antipsychotic drugs. Prevalence rates and degrees of severity of hyperprolactinemia (HPRL) based on PRL concentration may depend on the presence of macroprolactin in the serum. The aim of the study was to investigate the difference between PRL concentrations before and after precipitation of macroprolactin and to examine if there were any changes in the categorization of HPRL between samples prior and after precipitation.

**Materials and methods:**

Total of 98 female patients (median age 33; range 19-47 years) diagnosed with a psychotic disorder, proscribed antipsychotic drugs, and with HPRL were included. Total PRL concentration and PRL concentration after macroprolactin precipitation with polyethylene glycol (postPEG-PRL) were determined by the chemiluminometric method on the Beckman Coulter Access2 analyser.

**Results:**

Total PRL concentrations (median 1471; IQC: 1064-2016 mlU/L) and postPEG-PRL concentrations (median 1453; IQC: 979-1955 mlU/L) were significantly correlated using intraclass correlation coefficient for single measurements (mean estimation 0.96; 95%CI 0.93-0.97) and average measurement (mean estimation 0.98; 95%CI 0.96-0.99), and all investigated female patient had HPRL according to PRL and postPEG-PRL concentration. The median PRL recovery following PEG precipitation was 95; IQC: 90-100%. There was substantial agreement (kappa test = 0.859, 95% CI: 0.764-0.953) between the categories of HPRL severity based on total PRL concentrations and postPEG-PRL concentrations.

**Conclusion:**

The study demonstrated that HPRL was present in all subjects using the reference interval for total PRL concentration and postPEG-PRL concentration with no significant impact of macroprolactin presence in the serum on the categorization of patients according to severity of HPRL.

## Introduction

Hyperprolactinemia (HPRL) may be caused by various systemic conditions (pituitary disorders, advanced liver dysfunction, cirrhosis, chronic renal failure) and medications, including antidepressants, H2-antagonists, opioids and oestrogens (*1*). Hyperprolactinemia is also a common side effect of many antipsychotic drugs used for schizophrenia and bipolar disorder treatment (*2*–*4*). Antipsychotic drugs-induced HPRL has been estimated to occur in up to 70% of patients with schizophrenia (*3*, *4*). In addition, HPRL may have direct and indirect effect on the hypothalamic–pituitary–gonadal system, resulting in significant short-and long-term clinical consequences (*1*, *3*).

Patients with or without symptoms of HPRL may have high serum prolactin (PRL) concentrations, but prevalence rates and degrees of severity of HPRL may differ depending on the affinity of the antipsychotic drugs for D2 receptors, different penetrability across the blood-brain barrier and the modulation of monoamines other than dopamine (*5*).

According to Serri *et al*., the severity of HPRL in women may be defined as marked HPRL with PRL concentration > 2127 mlU/L (normally < 543mlU/L), commonly associated with hypogonadism, galactorrhoea and amenorrhea; moderate HPRL (1085-1595 mlU/L) associated with oligo menorrhea; mild HPRL (659-1063 mlU/L) associated with short luteal phase, decreased libido and infertility (*6*). In men, HPRL is associated with decreased libido, impotence, decreased sperm production, infertility, gynecomastia and, rarely, galactorrhoea, with the severity of HPRL differently than for females (*6*). Such proposed definition of HPRL severity was used in several guidelines for the management of antipsychotics induced HPRL (*7*–*10*).

These guidelines recommend a cut-off value for PRL concentration with a general notion that future action is needed to set protocols in relation to severity of HPRL and the presence of symptoms of HPRL (*7*–*10*). The first guideline set a cut-off for PRL concentration on 1000 mlU/L and included treatment guidelines (*7*). Another guideline, suggested determining pre-treatment PRL concentration, setting clear cut-off points for severity of HPRL, and deciding on the treatment for PRL concentrations > 1060 mlU/L, and PRL concentrations > 2120 mlU/L, even with no HPRL symptoms present, emphasizing the frequency in PRL monitoring in regard to the degree of HPRL (*8*). Some guidelines further suggests that PRL concentration should be determined after 3 months of receiving a stable dose of antipsychotic therapy or 3 months after changing the dose or the type of the therapy; repeating the test is recommended, with no consensus regarding frequency of this procedure, except for the need to test earlier if symptoms of HPRL present (*9*). In addition to the type of antipsychotic, the severity of HPRL depends on gender, age, length of follow-up and time of sampling, but in the studies on antipsychotics induced HPRL, the severity of HPRL has rarely been shown (*11*). However, other serum PRL isoforms may be the cause of an increase in the PRL concentration if there is no other apparent cause of HPRL, which may further affect the severity of HPRL (*12*).

Besides the 60-90% of circulating monomeric PRL (mPRL), 15-30% of covalently bound dimer (“big PRL”) and 0-10% larger polymeric form (“big- big” PRL) or macroprolactin (MPRL), are found in serum as well (*13*).

The predominance of MPRL above 30% or 60% (depending on the methodology used for detection of MPRL) is defined as macroprolactinemia (*13*–*15*). The prevalence of macroprolactinemia in the general population ranges between 3% and 4%, and increases to 35% in patients with HPRL (*12*), depending on the methodology used for detection and the population studied (*13*, *15*).

There is compelling evidence for the routine screening of hyperprolactinemic serum for MPRL (*2*). Some guidelines recommend the determination of the MPRL presence in asymptomatic patients, especially if the aetiology of HPRL is unclear (*2*, *16*). Similarly, specific guidelines for the management of antipsychotic drugs-induced HPRL suggest determining pre-treatment PRL concentration, and in case of the HPRL, MPRL screening (*9*). Determining MPRL is not recommended for patients with symptoms or with a known aetiology of HPRL (*2*, *16*).

The aim of the study was to investigate the difference between PRL concentrations before and after precipitation of macroprolactin and to examine if there were any changes in the categorization of HPRL between samples prior and after precipitation. The hypothesis of the study was that the severity of HPRL is related to the presence of MPRL in serum.

## Materials and methods

### Study design

The present retrospective cross-sectional study used the medical data from the laboratory information system in the Laboratory of Psychiatric Hospital Sveti Ivan (Zagreb, Croatia) in the period between September 2018 and March 2019. The total of 230 patients were analysed for PRL concentration in the given period. Exclusion criteria were set as following: we excluded patients whose PRL results were within and below the lower limit of the reference range suggested by the reagent manufacturer (71-566 mlU/L), male patients, female patients older than 50 years and patients with diagnoses other than the diagnosis of psychotic disorders who were on antipsychotic therapy. The results of repeated measurements for the same patients were excluded. The Hospital Ethical Committee approved the study.

### Subjects

The final sample included 98 female patients between 18 and 50 years (median 33 years; range 19–47 years), with PRL concentrations above the reference interval, admitted for treatment of psychotic disorders (psychotic disorder from the F20-F29 spectrum according to the ICD-10 classification) and taking antipsychotic drugs. Out of the 98 patients, 29 were on risperidone, 25 on paliperidon, 24 on clozapine, 13 on olanzapine, 20 on aripiprazole, five on haloperidol and three on quetiapine (as a monotherapy or a combination of antipsychotics).

### Blood sampling

All in-patients undergo routine blood screening test when admitted to the hospital. Blood specimens are extracted by phlebotomy procedure according to the national recommendations for venous blood sampling by the Croatian Society of Medical Biochemistry and Laboratory Medicine into the one vacuum serum test tube (6 mL) without anticoagulant (BD Vacutainer, Becton, Dickinson and Company, Franklin Lakes, USA) from the patients on the fasting state in the morning, before taking therapy (*17*). Given that PRL has daily fluctuations, reaching its highest value at waking hours, and being influenced by physiological factors like stress, exercise or meals, the blood specimen was extracted 1-2 hours after patient waking up after a minimum of a 30-minute patient resting period according to literature recommendation (*1*, *13*).

The blood specimen was left to rest at room temperature for one hour after the collection, following the manufacturer’s recommendation. After that period, it was centrifuged for 10 minutes on room temperature, at 2500xg. This serum sample was used to determine the concentration of total PRL immediately after MPRL precipitation (postPEG-PRL concentration).

The postPEG-PRL concentration was obtained after the precipitation of MPRL from serum sample, with 25% polyethylene glycol (PEG) 6000 solution (Merck, Hohenbrunn, Germany) prepared in deionized water. Once prepared, the solution was stored at 4 °C for a maximum period of three months. The process included adding 200 µl of 25% PEG solution to 200 µl serum and after 1 minute thorough vortex mixing, centrifuged at 1500xg for 30 min at 4 °C according to literature data (*18*, *19*). The supernatant was transferred to a new tube and analysed concentration of PRL in the supernatant and in the serum sample. PostPEG-PRL concentrations measured in supernatant were adjusted by factor 2 to correct for dilution in preparation.

### Methods

The PRL concentrations were measured using the Access 2 immunoassay analyser system (Beckman Coulter, Brea, USA) following the chemiluminescence immunoassay method, using the Access Prolactin reagents (lot number 771108; 871154). The test was calibrated with Access prolactin assay calibrators traceable to WHO 3rd IRP 84/500 standard (lot number 724012; 831821).

Immunoassay method is widely used but unable to differentiate mPRL from MPRL with different level of interferences (*15*, *20*). The gold standard for detecting of MPRL in serum is gel filtration chromatography (GFC), which is a slow and expensive method. The most widely used methodology is treating the hyperprolactinaemic serum with PEG solution, which precipitates out high-molecular weight isoform, including immunoglobulins, and re assay PRL concentration in supernatant (postPEG-PRL) with immunoassay method (*17*, *18*, *20*). This method has been validated against GFC with a different cut-off for the definition of macroprolactinemia (*14*, *18*-*22*). Potential misinterpretation was observed in patients with increased concentration of both mPRL and MPRL (*22*). Therefore, we used two ways to interpret the results: the percentage of PRL recovery (%REC) and using the reference interval for postPEG-PRL concentration. Using a reference interval for postPEG-PRL concentration is more useful, since expression of the %REC can be problematic if macroprolactinemia is present with HPRL (*23*).

A %REC was derived for each serum total PRL concentration as a percentage of the PRL concentration in the supernatant relative to the total PRL concentration in the untreated serum. The study used arbitrary cut-off values for %REC higher than 80% (as clinically insignificant MPRL content), 60–80% recovery as low MPRL content and less than 60% recovery as moderate to high MPRL content (*13*), and reference interval for postPEG-PRL concentration (92-469 mlU/L for females), specifically derived for Access assay for macroprolactinemia screening (*23*). Reference interval suggested by the manufacturer for total PRL concentration, and postPEG-PRL concentration was verified according to CLSI recommendation protocol EP28/A3c (*24*). The measured coefficient of variation was 2.10% for the concentration of control material at 145.7mlU/L (Lypocheck Immunoassay L1, BioRad, lot 40331) and 2.25% for the concentration of control material at 413.6 mlU/L (Lypocheck Immunoassay L2, BioRad, lot 40332).

### Statistical analysis

Normality of distribution for continuous variables was tested with Kolmogorov-Smirnov test. Median and interquartile range (IQC) were used as measures of central tendency and variability since most of the data were not distributed normally. Wilcoxon matched pairs signed ranks test was using for comparison between total PRL concentration and postPEG-PRL concentration. The intra class correlation coefficient (ICC) based on absolute agreement with a two-way model was used to determine the degree of correlation and agreement between the measurement of PRL concentration before and after precipitation. Mean estimation along with 95% confidence interval (CI) was reported for single measurements and average measurements. The interpretation was as follows: < 0.50 poor; > 0.50 and < 0.75, fair; > 0.75 and < 0.90 good; > 0.90 excellent. The percentage of recovery (%REC) was calculate as a percentage of the postPEG-PRL concentration in the supernatant relative to the total PRL concentration in the serum (%REC = 100 x total PRL/postPEG-PRL). The agreement between the categories of HPRL severity based on total PRL concentrations and postPEG-PRL concentrations was presented using the Weighted Kappa test (κ) for agreement. The 95% confidence intervals for Kappa statistic were given. Kappa statistic < 0.0 - 0.20 was considered slight agreement; 0.21 - 0.40 fair agreement; 0.41 - 0.60 moderate agreement; 0.61 - 0.80 substantial agreement, and 0.81 - 1.00 almost perfect agreement. The categories of HPRL severity according to serum total PRL concentration are defined as follows: mild 566-1000 mlU/L, moderate 1001-2000 mlU/L and severe > 2000 mlU/L. Level of significance was set to 95% (P < 0.05). The statistical data analysis was performed using Statistical Package for the Social Sciences (SPSS) version 23.0. (IBM, Armonk, NY).

## Results

Statistically significant difference between the total PRL concentration (median 1471; IQC: 1064-2016 mlU/L) and the postPEG-PRL concentration (median 1453; IQC: 979-1955 mlU/L) was obtained using the Wilcoxon matched pairs signed ranks test (Z = 5.06; P < 0.001). The ICC based on the absolute agreement between PRL concentration before and after precipitation was excellent for single measurements (mean estimation 0.96; 95%CI: 0.93-0.97) and average measurement (mean estimation 0.98; 95%CI: 0.96-0.99). The median and IQC for %REC following PEG precipitation was 95 (90-100) %. Recovery value > 80% was obtained for 95/98 female patients, 1/98 patient had %REC between 60-80%, 2/98 patients had %REC value < 60%. Total PRL concentration, postPEG PRL concentration and %REC for all participants were presented in the Table 1.

**Table 1:** Age, total PRL concentration, postPEG-PRL concentration and PRL percentage recovery for all included female patients

	**N**	**Median**	**IQC**
Age (years)	98	33 (19-47)	/
Total PRL-concentration, mlU/L	98	1471	1064-2016
post-PEG-PRL concentration, mlU/L	98	1453	979-1955
%Recovery	98	95	90-100
Age is presented as median (min-max). Total PRL concentration – prolactin concentration in untreated serum. PostPEG-PRL concentration – prolactin concentration after macroprolactin precipitation. %Recovery – prolactin percentage recovery after macroprolactin precipitation. N – number of female patients. IQC - interquartile range.			

**Figure 1:**
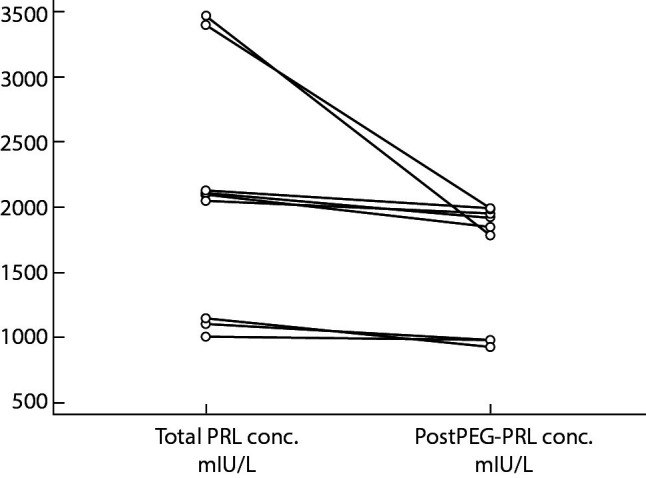
Differences between total PRL concentration and postPEG-PRL concentration and impact on the categorization of severity of HPRL (mild 566-1000 mlU/L, moderate 1001-2000 mlU/L and severe > 2000 mlU/L). Total PRL conc. – prolactin concentration in untreated serum. PostPEG-PRL conc – prolactin concentration after macroprolactin precipitation.

The categorization of patients according to severity of HPRL based on total PRL concentration and postPEG-PRL concentration were presented in the Table 2. There was substantial agreement between categorization of HPRL severity based on total PRL concentrations and postPEG-PRL concentrations (kappa test = 0.86; 95% CI: 0.76 to 0.95). However, nine patients were classified differently, but these patients had the total PRL concentration and postPEG-PRL concentration close to the cut-off value for individual category of severity of HPRL except for two patients with total PRL concentration above 3000 mlU/L (Figure 1).

**Table 2:** Agreement between categorization of HPRL severity based on total PRL concentration and postPEG-PRL concentration

	**Total PRL concentration, mlU/L**			**N**
PostPEG-PRL concentration, mlU/L	566-1000	> 2000	1001-2000	
566-1000	22	0	3	25
> 2000	0	22	0	22
1001-2000	0	6	45	51
N	22	28	48	98
Total PRL concentration – prolactin concentration in untreated serum; PostPEG-PRL concentration – prolactin concentration after macroprolactin precipitation; Kappa coefficient = 0.86 (95% CI: 0.76 to 0.95). N - number of participants.				

## Discussion

Only two patients had macroprolactinemia with HPRL suggesting that this type of HPRL induced by antipsychotic drugs is not influenced by the presence of macroprolactin in serum sample that was also shown in previous studies (*25*, *26*).

While a variety of different recovery cut-offs has been used to classify patients as having macroprolactinemia using a different methodology for PRL detection, we used recovery cut-off and postPEG-PRL reference interval for female participants derived specifically for the Access assay (*14*, *15*, *22*, *23*).

The study by Park *et al.* (*27*) found macroprolactinemia in 20.8% of the study population, but used a different method for MPRL determination and different cut-off value for macroprolactinemia definition (the macroprolactinemia was defined as a macroprolactin/total prolactin ratio with a cut-off at >30% using ELISA assay to determine MPRL concentration) which may have led to the discrepancy in findings with the present research.

The categorization of patients according to the severity of HPRL based on total PRL concentration and postPEG-PRL concentration were in substantial agreement (Table 2). For a minority of patients, MPRL may have little impact on the further treatment and diagnosis according to severity of HPRL because the values obtained for PRL and postPEG-PRL concentration are close to cut-off values for individual categories (Figure 1).

Two patients had a %REC < 60% with total PRL concentration greater than 3000 mlU/L. For them, MPRL concentration may have impact on the severity of HPRL, which may have a following impact on the patient’s treatment.

The present study found that the distribution of categories according to the HPRL severity based on the total PRL concentration is in line with the previously reported results shown by Bushe *et al*. for female patients with HPRL (included only female patients with the results of PRL concentration above reference range) (*28*, *29*). The present study results demonstrated that almost half of the subjects were categorized as having moderate HPRL based on total PRL concentration and postPEG-PRL concentration (Table 2.), as shown in the studies by Bushe *et al*. (*28*, *29*). The categorization of HPRL severity based on total PRL concentration in our study was adapted to the categorization of HPRL severity presented in the literature data by Serri *et al*. which is equally to the studies of Bushe *et al.* (*6*, *28*, *29*).

According to the literature data, most PRL concentration induced by antipsychotic therapy are between the upper limits of reference interval and 2120 mIU/L, but values greater than > 3180 mIU/L are possible, which were obtained also in our research according to total PRL concentration and postPEG-PRL concentration (*8*, *30*). However, the PRL values up to 2000 mlU/L may be due other pharmacotherapy, oestrogens, functional causes, or microprolactinomas, while macroadenomas are associated with concentrations over 5000 mlU/L (*30*).

The present study is important because it informs clinicians about the features of PRL assay used as well as for macroprolactinemia frequency, concerning the categorization of patients according to the severity of HPRL based on the total PRL concentration and postPEG-PRL concentration in patients with antipsychotics-induced HPRL. Consequently, MPRL screening seems to be necessary for some patients with antipsychotics-induced hyperprolactinemia with PRL concentration > 3000 mlU/L. Future research should further investigate the need for macroprolactinemia screening for this group of patients. Limitation of the study was relatively small sample size, especially for groups of female patients with the HPRL severity 566-1000 mlU/L and > 2000 mlU/L. However, these results are consistent with other available data showing similar categorization of female patients according to severity of HPRL based on total PRL and postPEG-PRL concentration.

In conclusion, the study demonstrated that HPRL was present in all subjects using the reference interval for total PRL concentration and postPEG-PRL concentration with no significant impact of MPRL presence in the serum on the categorization of patients according to severity of HPRL
